# Electrocardiographic Characteristics of Escape Rhythm During Complete Atrioventricular Block After Transcatheter Aortic Valve Replacement

**DOI:** 10.31083/RCM43915

**Published:** 2026-01-12

**Authors:** Itamar Loewenstein, Oren Yagel, Maayan Shrem, Daniel Lichtenstein, Gabby Elbaz-Greener, Oholi Tovia-Brodie, Jeremy Ben-Shoshan, David Planer, Yoav Michowitz, Maayan Konigstein, Bernard Belhassen

**Affiliations:** ^1^Department of Cardiology, Tel-Aviv Sourasky Medical Center, 64239 Tel Aviv, Israel; ^2^Heart Institute, Hadassah Medical Center, 91120 Jerusalem, Israel; ^3^Hadassah Hebrew University, 91120 Jerusalem, Israel; ^4^Jesselson Integrated Heart Center, Shaare Zedek Medical Center, 91031 Jerusalem, Israel; ^5^School of Medicine, Tel-Aviv University, 69978 Tel Aviv, Israel

**Keywords:** transcatheter aortic valve replacement, complete atrioventricular block, left bundle branch block, right bundle branch block, intra-Hisian block, escape rhythm

## Abstract

**Background:**

Complete atrioventricular block (CAVB) following transcatheter aortic valve replacement (TAVR) is primarily attributed to mechanical compression of the penetrating or branching portions of the His bundle, and less commonly, the atrioventricular (AV) node. This study aimed to characterize the electrocardiographic features of stable escape rhythms (ERs) occurring during CAVB after TAVR.

**Methods:**

This retrospective study analyzed 12-lead electrocardiograms (ECGs) obtained at three time points: before TAVR (ECG 1), after TAVR but before CAVB (ECG 2), and during CAVB (ECG 3). The ERs on ECG 3 were classified as AV junctional if the rate was 40–60 beats per minute (bpm) and, compared with ECG 2, if the QRS morphology matched in ≥10/12 leads, the QRS duration differed by <10 ms, and the frontal QRS axis differed by <30°. The ERs not meeting these criteria were considered ventricular in origin. Three patients with ERs <40 bpm but matching AV junctional morphology were included in the AV junctional group. ECG 2 was unavailable in 12 patients.

**Results:**

Among the 58 patients included, 56.9% had no conduction abnormalities on baseline ECG 1. Following TAVR (ECG 2), left and right bundle branch blocks were observed in 69.6% and 17.4% of the patients, respectively. During CAVB (ECG 3), the ERs were presumed to originate from the AV junction in 23 patients (39.6%), from the ventricles in 28 (48.3%), and had an undetermined origin in 7 (12.1%).

**Conclusions:**

Consistent with the anatomical regions commonly affected by the prosthetic aortic valve during TAVR, a substantial proportion of patients exhibited ERs likely originating from the AV junction, suggesting a potential role for conduction system pacing in managing CAVB in this setting of patients.

## 1. Introduction

Complete atrioventricular block (CAVB) occurs in approximately 15% of patients within 30 days following transcatheter aortic valve replacement (TAVR), particularly among those with pre-existing right bundle branch block (RBBB) or those who develop new-onset left bundle branch block (LBBB) post-procedure [1]. The underlying mechanism is primarily attributed to mechanical compression of the penetrating or branching portions of the His bundle, and less frequently, to injury of the atrioventricular (AV) node [1, 2, 3, 4].

While some cases necessitate immediate ventricular pacing, many patients with post-TAVR CAVB present with stable escape rhythms detected during routine electrocardiographic (ECG) monitoring. Characterization of these escape rhythms may provide insights into the anatomical site of conduction block.

In this study, we systematically evaluated the ECG features of stable escape rhythms during CAVB in patients undergoing TAVR. To the best of our knowledge, this represents the first study specifically addressing this phenomenon in the TAVR population.

## 2. Methods

### 2.1 Population Cohort

This is a retrospective study involving patients who underwent their first TAVR for severe aortic stenosis. The study was approved by the Institutional Research Ethics Board at Tel-Aviv Sourasky Medical Center and followed the ethical norms and standards in the Declaration of Helsinki. All patients undergoing TAVR in 3 Israeli institutions (Tel-Aviv, Hadassah, and Shaare Zedek Medical Centers) provided informed consent for the procedure as well as for the secondary use of clinical data for research. The data that support the findings of this study are available from the corresponding author upon reasonable request.

### 2.2 Study Group

Patients were eligible for inclusion if the following criteria were met:

(1) A baseline 12-lead ECG obtained prior to TAVR demonstrated 1:1 AV conduction during sinus rhythm or an irregular ventricular response during atrial flutter or fibrillation (ECG 1);

(2) At least one intermediate ECG recorded after TAVR but before the onset of CAVB was available (ECG 2). When multiple intermediate ECGs were present, the most recent recording prior to CAVB documentation was selected;

(3) A 12-lead ECG demonstrating CAVB with a stable escape rhythm lasting at least 10 seconds was obtained following TAVR (ECG 3). If a second stable escape rhythm with a different morphology was documented, it was also included in the analysis. Patients for whom only ECGs 1 and 3 were available were analyzed separately.

Patients with temporary or permanent pacemakers implanted before or after TAVR were not excluded, provided the devices were inactive at the time ECGs 1, 2, and 3 were recorded.

Demographic and clinical data were extracted from the hospitals’ electronic medical records, including hospitalization summaries and TAVR procedure reports. Procedural data included valve type.

### 2.3 ECG Analysis

Analysis of ECGs 1 and 2 included assessment of the underlying rhythm (sinus rhythm vs. atrial fibrillation/flutter), sinus rate (beats per minute), PR interval (milliseconds), QRS duration (milliseconds), frontal QRS axis (degrees), corrected QT interval (QTc, calculated using Bazett’s formula), and identification of any intraventricular conduction disturbances (IVCDs).

Analysis of ECG 3, recorded during CAVB, involved evaluation of the atrial rhythm (sinus vs. atrial fibrillation/flutter), escape rhythm rate, QRS morphology (classified as LBBB or RBBB), QRS duration, frontal QRS axis, and QTc.

All ECG measurements—including heart rate, PR interval, QRS duration, frontal axis, and QTc—were obtained using both manual and automated methods.

### 2.4 Definitions 

The AV junction includes the AV node, its penetrating portion, and the non-branching segment of the His bundle. The subjunctional region refers to the branching portion of the His bundle, extending from the origin of the first fibers of the LBB to the takeoff of the RBBB [5, 6].

The escape QRS rhythm observed on ECG 3 was defined as “similar” to the QRS complex on ECG 2 if the following criteria were met: (1) QRS morphology was comparable in more than 10 out of 12 leads; (2) QRS duration differed by no more than 10 milliseconds; and (3) the frontal QRS axis differed by no more than 30°. If ECG 2 was unavailable, the degree of similarity was recorded as “unknown”.

The mechanism of the escape rhythm during CAVB was classified as “AV junctional” if the heart rate was between 40 and 60 beats per minute [7] and the QRS complexes in ECGs 2 and 3 were similar in morphology, duration, and axis, as defined above. Additionally, three patients with escape rhythms meeting the morphological criteria for an AV junctional origin but with slower rates (28–34 beats per minute (bpm)) were also included in the AV junctional group.

Escape rhythms were classified as “ventricular” if the QRS morphology, duration, or axis differed between ECGs 2 and 3. In cases where ECG 2 was unavailable, ECG 3 was compared with ECG 1. If the QRS complexes in ECG 3 matched those in ECG 1, the escape rhythm was considered AV junctional in origin; otherwise, the origin was classified as “unknown”.

Unspecified IVCD, LBBB, RBBB, and frontal QRS axis were defined according to the AHA/ACCF/HRS Scientific Statement [8]. A normal QRS axis was defined as –30° to +90°, left axis deviation as –30° to –90°, and right axis deviation as +90° to +180°. Left fascicular blocks were classified according to the criteria proposed by Pérez-Riera *et al*. [9].

### 2.5 Statistical Analysis

Categorical variables are reported as numbers (%) and compared using Fisher’s exact test. Continuous variables are reported as medians (IQR) and compared using Mann-Whitney U tests, or as mean ± SD and compared using the student’s *t*-test. Analysis of variance (ANOVA) was used to compare means across multiple groups. Paired samples *t*-tests were used to compare different variables for the same patient. A two-tailed *p*-value less than 0.05 was considered statistically significant. Analyses were conducted using IBM SPSS (29.0, IBM Corp., Armonk, NY, USA).

## 3. Results

### 3.1 Baseline Demographic, Clinical, and Procedural Characteristics 

The study cohort included 58 patients who underwent TAVR procedures between August 2010 and May 2024, originating from three centers: 24 from Tel-Aviv Medical Center, 22 from Hadassah Medical Center, and 12 from Shaare Zedek Medical Center. The majority of patients were female (n = 35, 60.3%), with a mean age of 80.7 ± 6.5 years. The average body mass index (BMI) was 28.2 ± 5.2 kg/m^2^. A history of coronary artery disease, coronary artery bypass grafting (CABG), and prior aortic valve intervention was present in 24 (41.4%), 6 (10.3%), and 2 (3.4%) patients, respectively.

The median left ventricular ejection fraction was 60% (interquartile range [IQR] 55–60%). The mean peak aortic valve velocity was 5.69 ± 9.25 m/s, the mean aortic valve pressure gradient was 45.9 ± 17.5 mmHg, and the mean aortic valve area was 0.69 ± 0.18 cm^2^. Baseline clinical and echocardiographic characteristics were comparable across the three medical centers.

Most patients (n = 37, 63.8%) received a self-expandable valve (SEV), predominantly from the Medtronic company, while the remaining 21 patients (36.2%) received a balloon-expandable valve (BEV), primarily from the Edwards company.

### 3.2 Baseline ECG Characteristics (ECG 1)

**Fig. 1.**
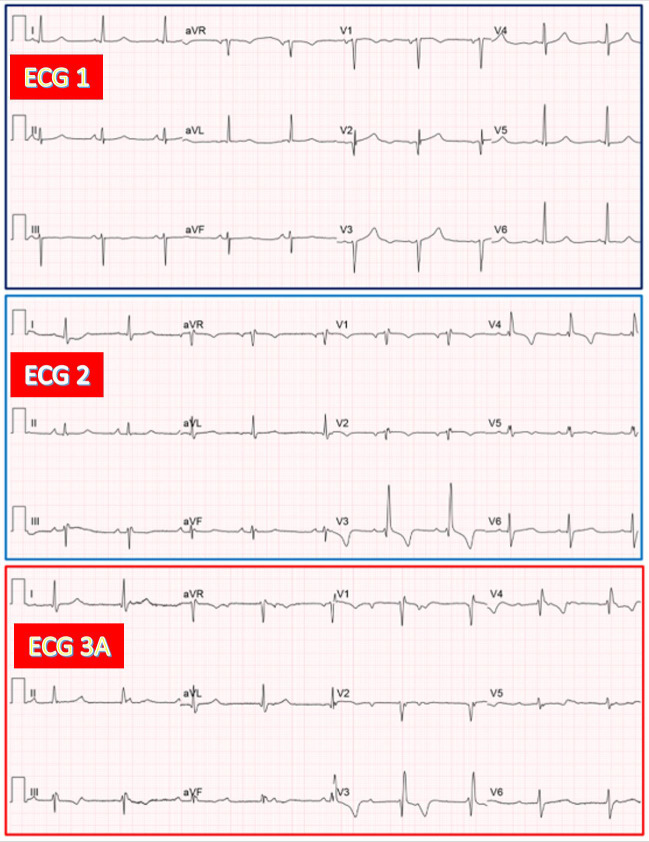
**AV junctional escape rhythm with RBBB during CAVB in an 83-year-old woman exhibiting RBBB after TAVR**. (ECG 1) Sinus rhythm with narrow QRS and poor R wave progression in V1-V3. (ECG 2) Sinus rhythm with slight PR increase and RBBB. (ECG 3A) Recorded 8 hours after TAVR. CAVB with AV junctional rhythm (53/min), having QRS morphology similar to that on ECG 2. AV, atrioventricular; RBBB, right bundle branch block; CAVB, complete atrioventricular block; TAVR, transcatheter aortic valve replacement; ECG, electrocardiographic.

**Fig. 2.**
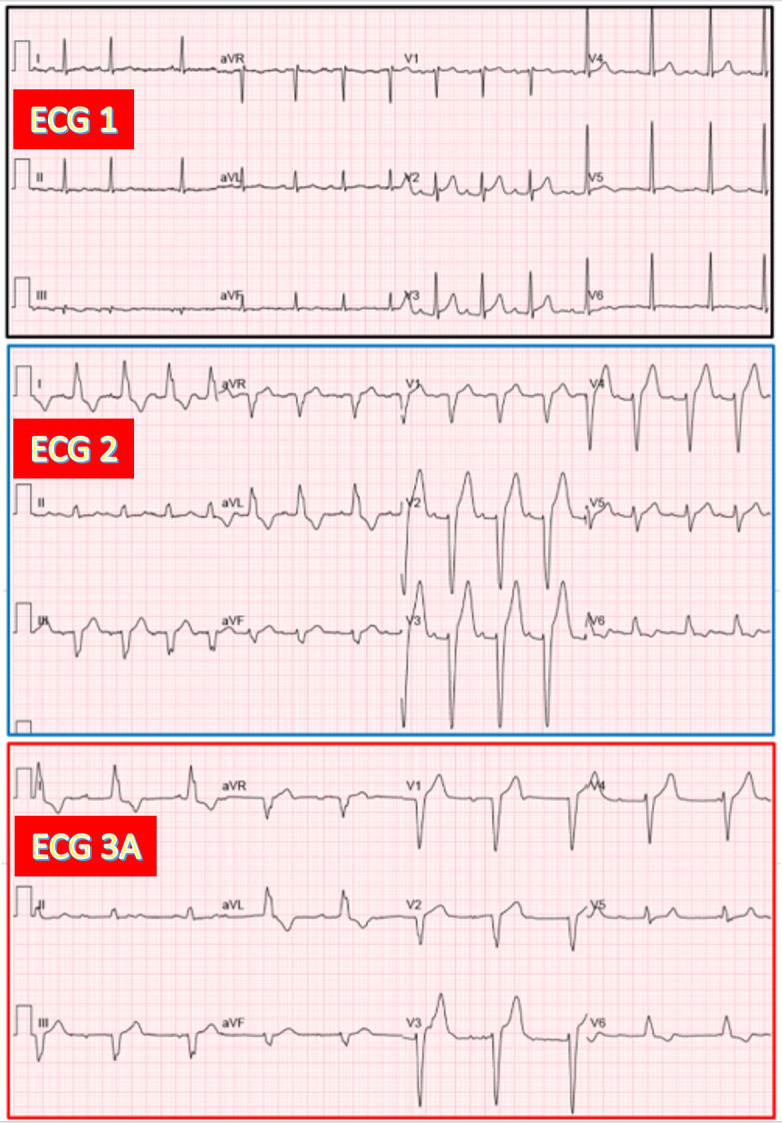
**AV junctional escape rhythm with LBBB during CAVB in an 81-year-old man exhibiting LBBB after TAVR. (**ECG 1) Sinus rhythm with narrow QRS, with slight ST elevation in V2-V3. (ECG 2) Sinus rhythm with PR increase and LBBB. Note two atrial extrasystoles in leads I-II-III. (ECG 3A) Recorded 40 hours after TAVR. CAVB with AV junctional escape rhythm (57/min) having QRS morphology similar to that on ECG 2. LBBB, left bundle branch block.

**Fig. 3.**
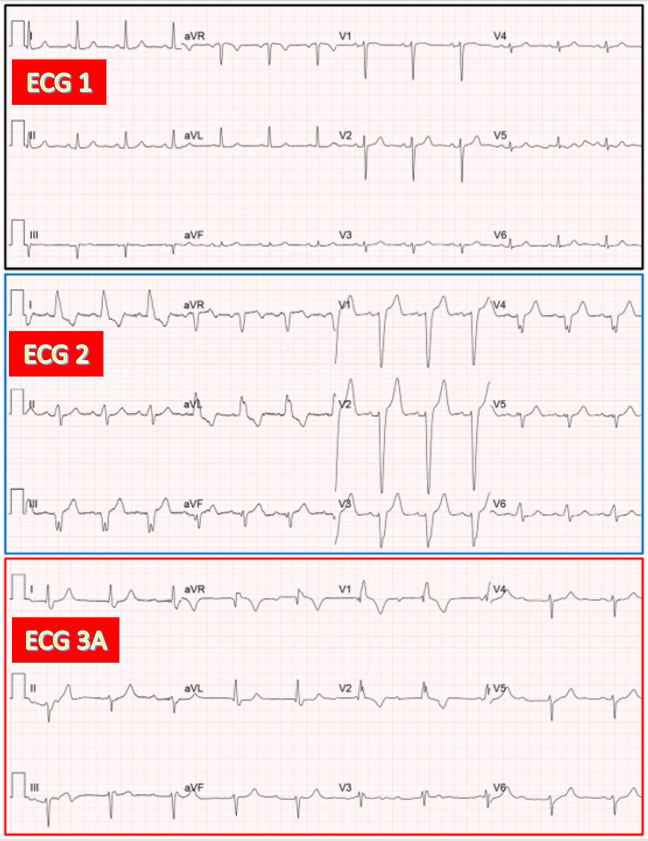
**Ventricular escape rhythm with RBBB pattern during CAVB in a 73-year-old woman exhibiting LBBB after TAVR**. (ECG 1) Sinus rhythm with narrow QRS. (ECG 2) Sinus rhythm with LBBB. (ECG 3A) Recorded 10 hours after TAVR. CAVB with ventricular escape rhythm (58/min), having an RBBB pattern.

**Fig. 4.**
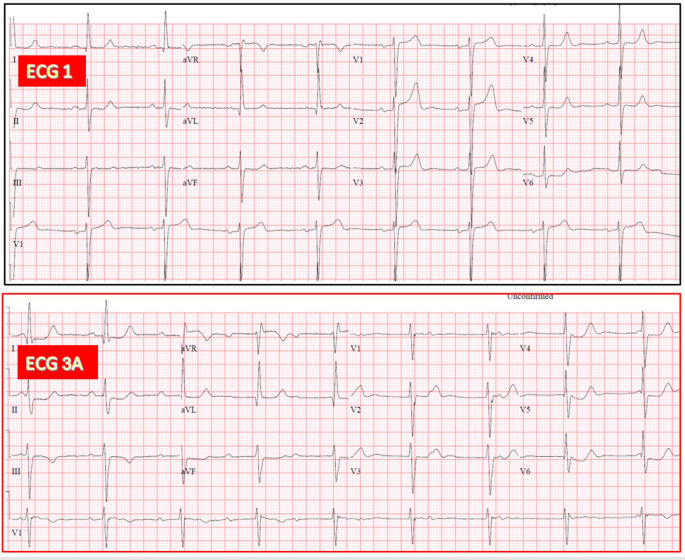
**Presumed AV junctional escape rhythm associated with narrow QRS complexes in an 83-year-old woman during CAVB after TAVR**. (ECG 1) Sinus rhythm with narrow QRS. (ECG 3A) recorded 24 hours after TAVR. CAVB with AV junctional escape rhythm (53/min) having QRS morphology similar to that on ECG 1.

**Fig. 5.**
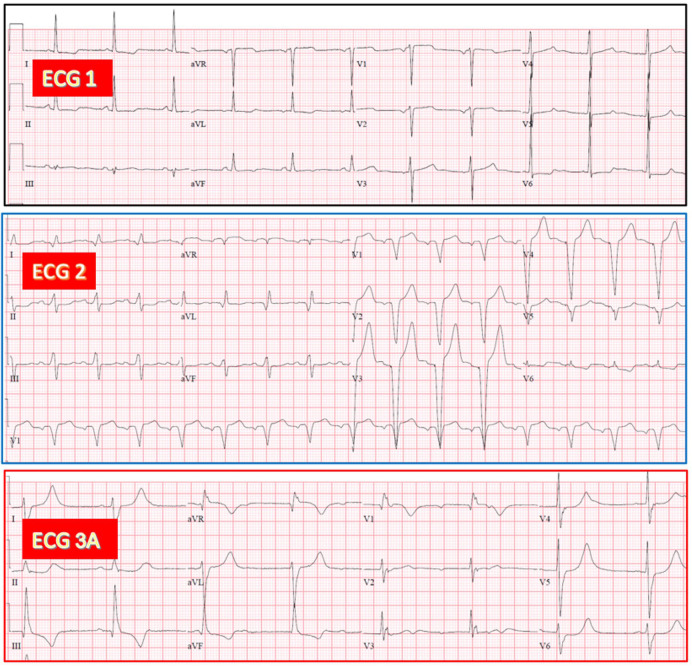
**Ventricular escape rhythm during CAVB in a 76-year-old woman developing LBBB after TAVR**. (ECG 1) Sinus rhythm with narrow QRS. (ECG 2) Sinus rhythm with PR increase and LBBB. (ECG 3A) Recorded 24 hours after TAVR. CAVB with ventricular escape rhythm (47/min) having RBBB + LPFB morphology. LPFB, left posterior fascicular block.

**Fig. 6.**
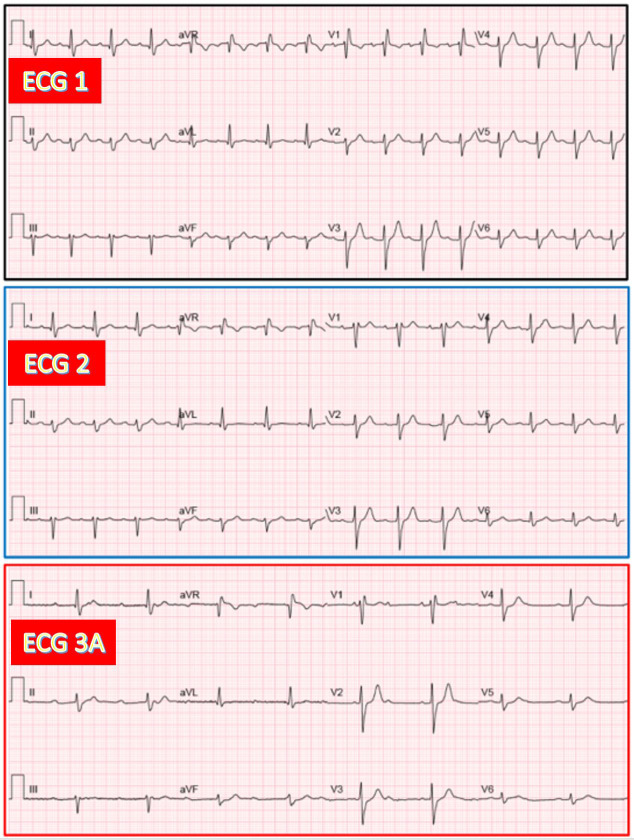
**AV junctional escape rhythm with RBBB and LAFB pattern during CAVB in a 73-year-old man exhibiting the same bifascicular block before and after TAVR**. (ECG 1) Sinus rhythm with PR prolongation and RBBB plus LAFB. (ECG 2) Sinus rhythm with a similar bifascicular block. (ECG 3A) CAVB with AV junctional rhythm (51/min) having QRS morphology similar to that on ECG 2.

**Fig. 7.**
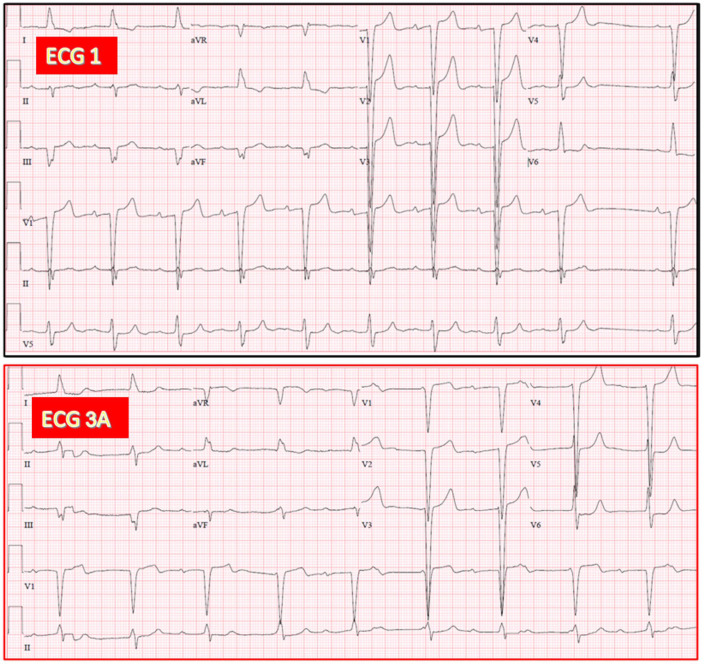
**AV junctional escape rhythm with LBBB during CAVB, similar to baseline LBBB in a 93-year-old woman after TAVR (no ECG 2 available)**. (ECG 1) Sinus rhythm with prolonged PR and LBBB. (ECG 3A) Recorded 24 hours after TAVR. CAVB with AV junctional escape rhythm (55/min), having QRS morphology similar to that on ECG 1.

**Fig. 8.**
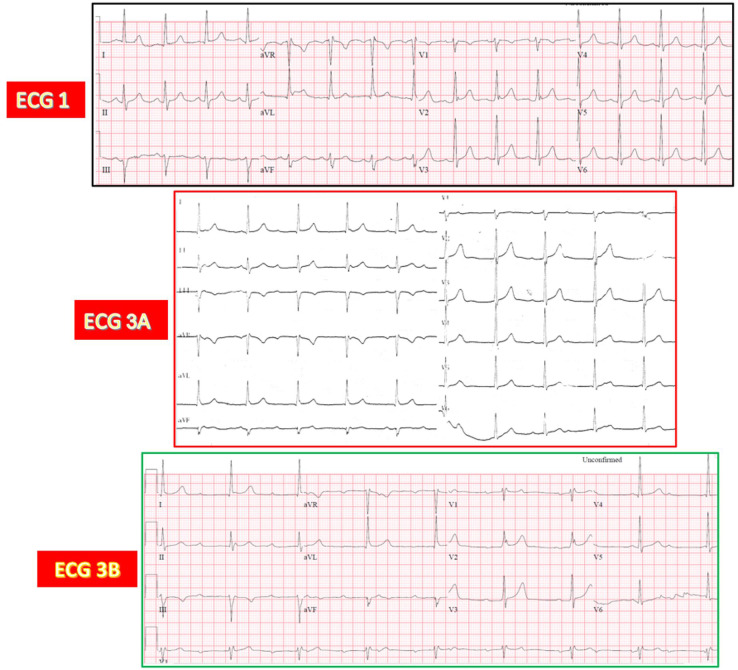
**AV junctional escape rhythm with narrow QRS complexes during CAVB in a 73-year-old man after TAVR (no ECG 2 available)**. (ECG 1) Sinus rhythm with suspected LSFB. (ECG 3A) Recorded 24 hours after TAVR. CAVB with AV junctional escape rhythm (60/min), having QRS morphology similar to that on ECG 1. (ECG 3B) Recorded 20 minutes after ECG 3A. CAVB with another slower escape rhythm (50/min), which exhibits a RBBB morphology in leads V1 and V2 as compared to ECG 3A, and was classified as having a presumed ventricular origin. LSFB, left septal fascicular block.

Sinus rhythm was present in all patients except one, with rates ranging from 46 to 92 (mean 68.7 ± 11.8) beats/min. Mean PR interval was 187.7 ± 45.8 milliseconds, and 19 patients had a PR interval >200 milliseconds. Mean QRS axis was –1.3 ± 38.6°, and the mean QTc interval was 447.8 ± 53.6 milliseconds. No IVCDs were observed in 33 patients (56.9%) (Figs. 1,2,3,4,5). RBBB was identified in 10 (17.2%) patients (associated with left anterior, posterior, or septal fascicular blocks in 4, 1, and 1 patients, respectively) (Fig. 6). LBBB was observed in 5 (8.6%) patients (Fig. 7). An unclassified IVCD, a left anterior fascicular block (LAFB), and a suspected left septal fascicular block (LSFB) were present in 3, 3, and 4 patients, respectively (Fig. 8).

### 3.3 ECG Characteristics After TAVR but Before CAVB (ECG 2)

Among the study population, ECG 2 was available in 46 patients (79.3%). Sinus rhythm was present in 42 (91.3%) patients, with mean PR intervals of 213.9 ± 46.3 milliseconds. Mean QRS axis was –20.5 ± 36.9° and mean QTc interval was 479.7 ± 32.3 milliseconds.

With the exception of one patient (2.2%), all ECG 2 recordings demonstrated an IVCD. The most frequently observed was LBBB, present in 32 patients (69.6%) (Figs. 2,3,5), followed by RBBB in 8 patients (17.4%), of whom 5 also had LAFB (Figs. 1,6). Other types of IVCD were identified in the remaining 5 patients (10.9%). Among the 28 study patients who did not show any IVCD on baseline ECG 1, and for whom ECG 2 was available, LBBB was the most frequently observed on ECG 2 (n = 25, 75.8%) (Figs. 2,3,5), followed by RBBB (n = 2, 6.1%) (Fig. 1), and LAFB in 1 patient.

**Fig. 9.**
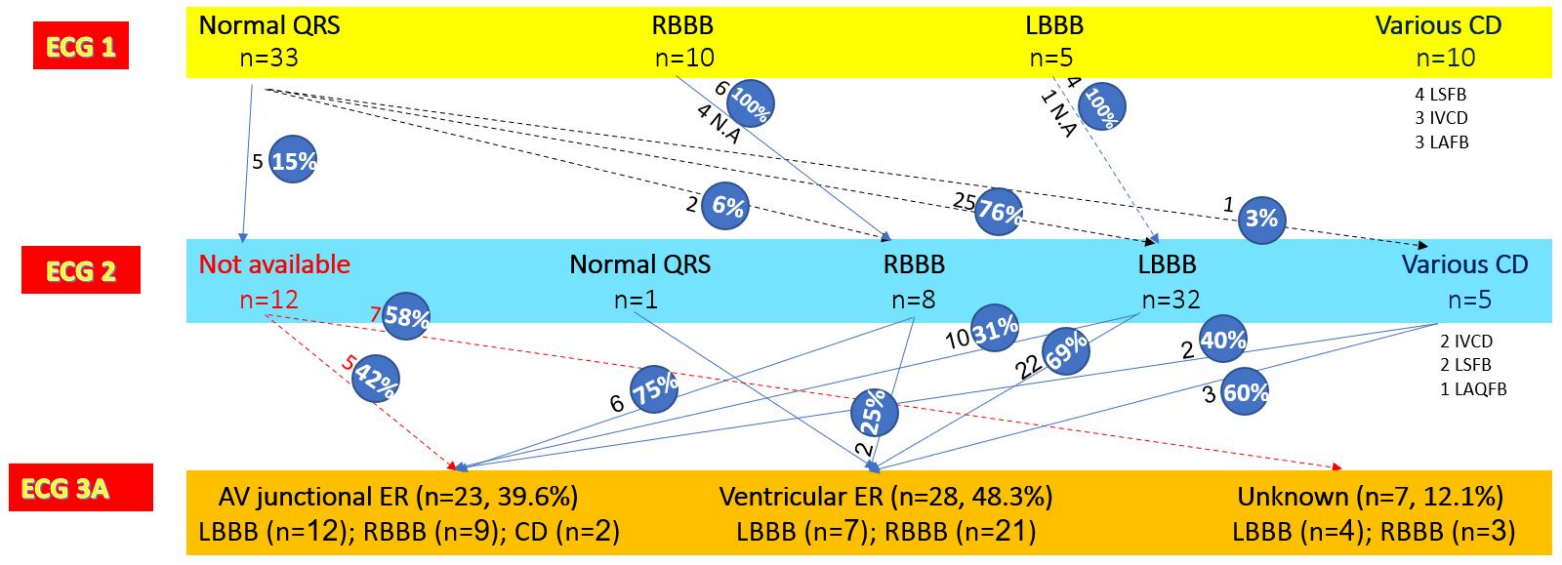
**Flowchart of the ECG findings before and after TAVR and during CAVB**. Abbreviations: AV, atrioventricular; CD, conduction disturbances; ER, escape rhythm; IVCD, undetermined intraventricular conduction disturbance; LAFB, left anterior fascicular block; LBBB, left bundle branch block; LSFB, left septal fascicular block; N.A, not available; RBBB, right bundle branch block.

The progression of conduction disturbances from ECG 1 to ECG 2 for all patients is illustrated in the flowchart (Fig. 9).

### 3.4 Number of Escape Rhythms Documented During CAVB

A single morphological type of escape rhythm (ECG 3A) was documented in 50 patients (86.2%), while a second, distinct escape rhythm (ECG 3B) was observed in the remaining 8 patients (13.8%). No additional escape rhythm types were recorded.

### 3.5 ECG Characteristics of the First Escape Rhythm During CAVB (ECG 3A)

In 46 patients (79.3%), the QRS escape rhythms observed during CAVB on ECG 3 could be compared with the supraventricular rhythm (sinus or atrial fibrillation) recorded on ECG 2. In the remaining 12 patients, such a comparison was not possible due to the absence of ECG 2. After excluding these 12 patients, and based on the similarity in QRS morphology, duration, and axis, the first escape rhythm was presumed to have an AV junctional origin in 18 (31%) study patients (Figs. 1,2,6) and a ventricular origin in 28 (48.3%) (Figs. 3,5).

Of the 18 patients in whom the escape rhythm was presumed to have an AV junctional origin, the QRS morphology in ECG 2 had an LBBB pattern in 10 (55.6%) patients (Fig. 2) and a RBBB pattern in 6 (33.3%) patients (associated with LAFB in 4 patients) (Figs. 1,6); in the remaining 2 patients, various conduction disturbances were present (undetermined IVCD in 1 patient and incomplete RBBB plus LAFB in another one).

Among the 28 patients whose escape rhythm was presumed to be of ventricular origin, the QRS morphology in ECG 2 had an LBBB and RBBB morphology in 22 patients (Figs. 3,5) and 2 patients, respectively; in the remaining 4 patients, various conduction disturbances were present.

In 5 of 12 patients without an available ECG 2 (representing 8.6% of the study population), the escape rhythm observed on ECG 3 was similar to that on ECG 1 and was therefore classified as having an AV junctional origin. Among these 5 patients, the baseline QRS complex was normal in 2 cases (Fig. 4) while the remaining 3 exhibited LBBB (Fig. 7), suspected LSFB (Fig. 8), and RBBB associated with left posterior fascicular block (LPFB), respectively. In the remaining 7 patients who had no ECG 2 available (12.1% of the study patient group), the escape mechanism was classified as unknown.

The progression of conduction disturbances from ECG 2 to ECG 3A (first documented escape rhythm) is illustrated in the flowchart (Fig. 9).

### 3.6 ECG Characteristics of the Second Escape Rhythm During CAVB (ECG 3B) 

Of the 58 patients, 8 (13.8%) had a second escape rhythm that was markedly different from the first escape rhythm. All the second escape rhythms were classified as having a ventricular origin; none was classified as having an AV junctional origin.

**Fig. 10.**
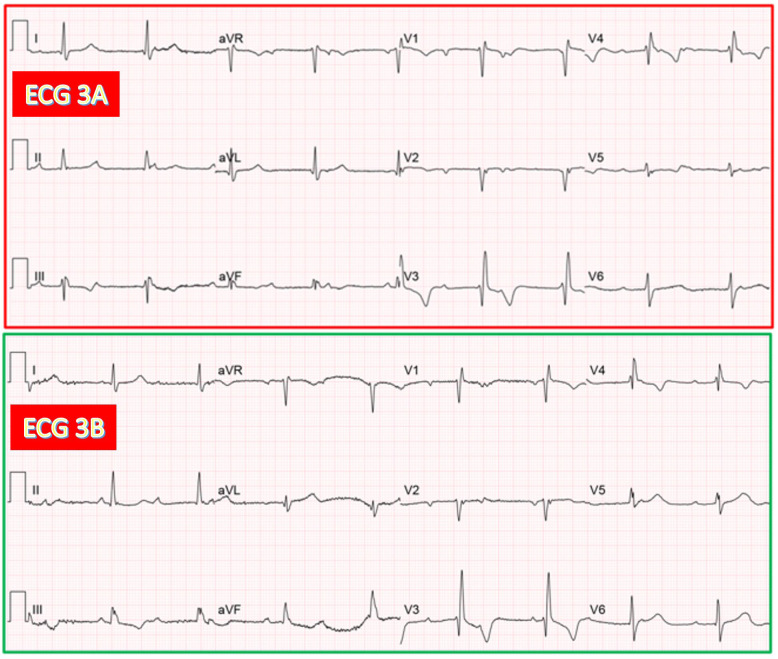
**Same patient as in Fig. 1. Documentation of both AV junctional and ventricular escape rhythms after TAVR**. (ECG 3A) Recorded 4 hours after TAVR. CAVB with AV junctional rhythm (53/min) having RBBB morphology. (ECG 3B) Recorded 24 hours after TAVR. CAVB with presumed ventricular escape rhythm (51/min) due to a marked difference in morphology with the previous AV junction rhythm in limb leads (relative QRS axis shift to the right). Interestingly, the last QRS complex in leads V4-V6 looks slightly different in morphology (especially in V4-V5) from the previous one and more similar to the escape rhythm in the same ECG leads on ECG 3A.

In the 8 patients having 2 types of escape rhythms documented (Fig. 10), the mean rates of the escape rhythms were 54.9 ± 4.8 and 53.6 ± 8.8 beats/min (*p* = 0.68), the mean QTc intervals were 477.4 ± 32.1 and 468.1 ± 44.2 milliseconds (*p* = 0.35) and the mean QRS durations were 129.4 ± 17.1 and 123.4 ± 14.3 milliseconds (*p* = 0.38) for escapes #1 and #2, respectively.

### 3.7 Comparison of Rates, QRS Duration, QTc, and Patterns of Escape Rhythms in Respect to Their Presumed Site of AVB

The mean rate of escape rhythms presumed to have an AV junctional origin (n = 23) or a ventricular origin (n = 28) were 50.4 ± 9.8 and 47.9 ± 9.0 beats/min, respectively (*p* = 0.34); the mean QRS durations were 135.1 ± 16.7 and 141.5 ± 14.8 milliseconds, respectively (*p* = 0.152) while the mean QTc values were 459.0 ± 46.4 and 463.6 ± 52.3 milliseconds, respectively (*p* = 0.746). For patients with AV junctional escape rhythms, an LBBB pattern occurred in 12 (52.2%) cases, an RBBB pattern in 9 (39.1%), and two patients had a non-specific escape pattern. Of the 28 patients having a ventricular escape rhythm, an LBBB escape pattern was prevalent in 7 (25%) cases and an RBBB pattern in 21 (75%). In the latter group, normal QRS axis, left axis, and right axis were present in 9, 8, and 4 patients, respectively.

For both patients with presumed junctional or ventricular escape rhythms, escape rates were significantly slower than baseline sinus rhythm rates, and following TAVR, QRS durations increased significantly compared to pre-TAVR values, with no significant difference observed between post-TAVR and escape rhythm QRS durations. QRS axis exhibited significant deviation after TAVR in both groups, while additional significant shifts between post-TAVR and CAVB recordings occurred exclusively in patients with ventricular rhythms. QTc intervals demonstrated primary prolongation following TAVR, with heterogeneous findings in the third recording across comparisons.

### 3.8 Mechanism of First Escape Rhythm With Respect to Demographics and TAVR Type

An AV junctional rhythm during CAVB was observed in 15 of 35 (42.9%) females and in 8 of 23 (34.8%) males (*p* = 0.54). The patients who had an AV junctional rhythm and ventricular rhythm during CAVB were aged 81.5 ± 6.2 and 80.3 ± 7.0 years old, respectively (*p* = 0.54). An AV junctional rhythm was observed in 13 of 30 (43.3%) patients who had an SEV implanted and in 10 of 21 (47.6%) patients who had a BEV implanted (*p* = 0.76). A ventricular escape rhythm was observed in 17 of 30 (56.7%) patients who had an SEV implanted and in 11 of 21 (52.4%) patients who had a BEV implanted (*p* = 0.76).

## 4. Discussion

Our study is the first, after more than 3 million TAVR procedures performed and hundreds of thousands of pacemakers implanted worldwide during the last 2 decades, which analyzes the ECG characteristics of escape rhythm during CAVB after TAVR in a relatively large series of patients. This is the first series assessing the demographic and procedural characteristics of patients exhibiting CAVB with stable escape rhythms after TAVR. In addition, this is the largest collection of 12-lead ECG recordings during CAVB in which a careful ECG analysis of the sequence of occurrence of AVB and the QRS morphology of the escape rhythm enables us to postulate on the anatomical site and pathophysiologic mechanism of the AVB for each patient.

### 4.1 Site of CAVB Following TAVR

CAVB after TAVR is explained by the proximity of the conduction system (especially the His bundle and its branching portion) to the prosthetic aortic valve deployed, following direct mechanical compression or an indirect injury by perivalvular inflammation or ischemia [1, 2, 3, 10]. The His bundle courses from the inferior margin of the membranous septum to the ventricular septal crest, near the junction of the right and noncoronary aortic cusps, making it particularly susceptible to injury during TAVR [10]. In rare cases, the AV node—which is situated near the aortic annulus—may also be affected [4]. To the best of our knowledge, only a single necropsy report has described a post-TAVR patient who developed CAVB requiring transvenous pacemaker implantation [3]. The presumed cause of death was right ventricular perforation due to the pacing electrode. Notably, a localized hematoma was identified at the interventricular septum, the site of aortic valve prosthesis expansion, which was found to compress the His bundle. Based on the escape rhythm characteristics observed during CAVB in our study, we estimate that the site of block was located in the AV junctional region in approximately 39.6% of patients.

Despite the large number of diagnostic electrophysiological studies (EPS) conducted in post-TAVR patients to assess the need for prophylactic pacemaker implantation, a comprehensive review of the literature revealed only two reports specifically addressing EPS findings in patients who developed CAVB following TAVR. In one study, Vijayaraman *et al*. [11] performed His bundle pacing in 100 patients with advanced AV block or CAVB, classifying the block as infranodal in 54% and intranodal in 46% of cases. Although ECG characteristics and the proportion of patients with intra-Hisian disease were not detailed, a representative tracing was presented from a patient with 2:1 AV block and RBBB, where both conduction disturbances were attributed to a proximal His bundle lesion.

In a subsequent report, the same group described a cohort of 65 TAVR patients who underwent attempted permanent His–Purkinje conduction system pacing [12]. Infranodal AV block was identified in 58 patients (89%), of whom 39 (67%) had persistent CAVB and 19 (33%) had intermittent or Mobitz II block. While the prevalence of intra-Hisian disease was not specified, the authors included a tracing from a patient with a 2:1 intra-His block and normal QRS duration that progressed to complete intra-His block during His bundle mapping [12]. Additional isolated reports of severe AV nodal involvement following TAVR have also been documented [4, 11].

In the absence of His bundle recordings in our study cohort, the precise site of CAVB cannot be definitively established. However, the presence of at least one escape rhythm consistent with an AV junctional origin in nearly 40% of patients suggests that the AV junctional area may have been affected by the prosthetic valve. Moreover, the absence of significant PR interval prolongation or Wenckebach-type second-degree AV block on ECG monitoring prior to the onset of CAVB [4] supports the notion that the primary site of injury was more likely the His bundle rather than the AV node.

### 4.2 Site of Origin of the Escape Rhythm

Likewise, the absence of His bundle recordings in our study population precludes definitive identification of the site of origin of the escape rhythms. Therefore, only speculative interpretations can be offered.

One possible explanation involves the concept of longitudinal dissociation within the His bundle, a phenomenon well documented in both human and animal studies for over a century [13, 14, 15, 16]. It has been proposed that LBBB—the most common conduction disturbance observed following TAVR—may result from selective injury to the left-sided fibers of a longitudinally dissociated His bundle [15, 16, 17, 18, 19, 20].

**Fig. 11.**
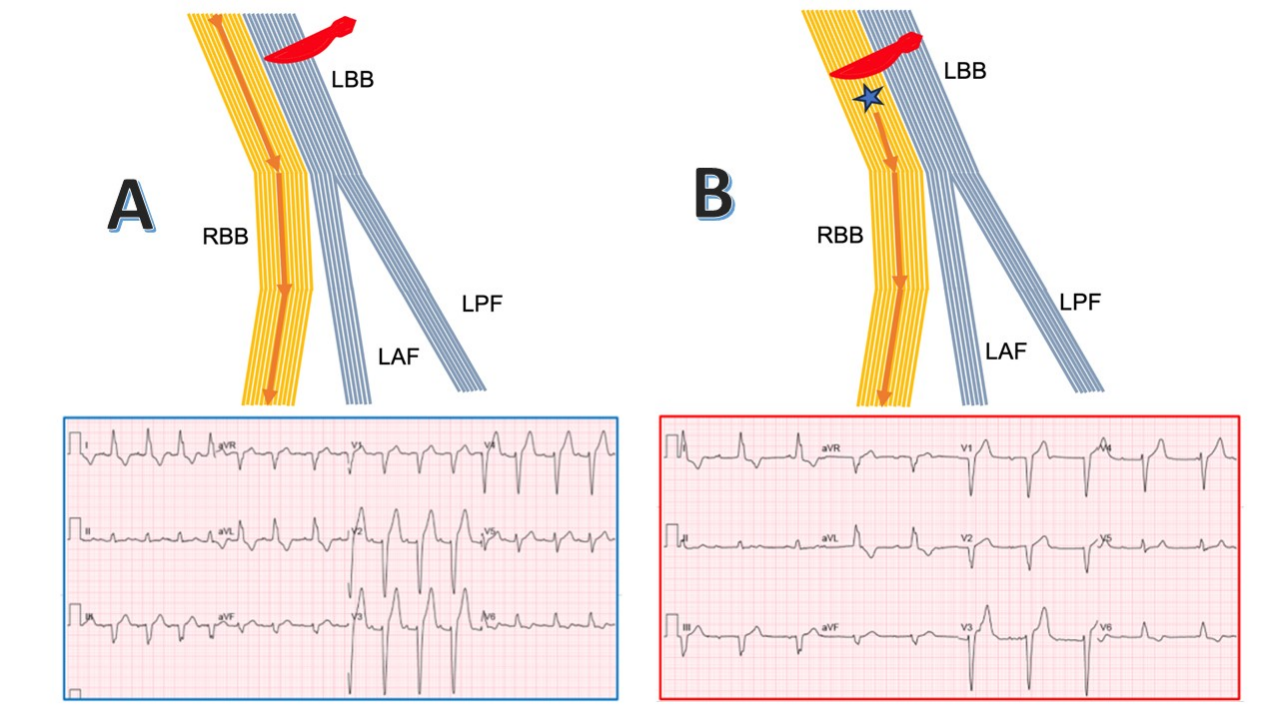
**Potential mechanism for AV junctional rhythm following TAVR-induced LBBB relying on the theory of longitudinal dissociation of the His bundle**. (A) LBBB is created by a lesion in the left-sided fibers of the His bundle (red scalpel). (B) An additional lesion in the right-sided fibers of the His bundle resulting in CAVB (red scalpel) and an AV junctional rhythm originating from below the RBB (blue asterisk). Abbreviations: LBB, left bundle branch; LAF, left anterior fascicle; LSFB, left septal fascicle; LPF, left posterior fascicle; RBB, right bundle branch.

Decades ago, Rosenbaum and colleagues [21], building on earlier findings by Singer *et al*. [22] in isolated Purkinje fibers, demonstrated that in both canine and human hearts, escape rhythms tend to emerge below the level of fascicular injury. Based on that, it is plausible to hypothesize that in patients with post-TAVR LBBB, further extension of the mechanical injury to the right-sided His fibers may result in CAVB, with the escape rhythm arising just below the site of injury—presumably within the AV junction. In such cases, the escape rhythm would display a QRS morphology similar to the LBBB pattern already present after TAVR (Fig. 11).

Similarly, in patients with RBBB prior to or following TAVR who then develop CAVB due to damage to the left-sided His fibers, the resultant escape rhythm would likely arise below the site of the latest injury and exhibit a QRS morphology consistent with RBBB—again resembling the pre-block conduction pattern.

Our findings support this hypothesis, with 12 patients exhibiting LBBB-pattern escape rhythms and 9 showing RBBB-pattern escape rhythms. An alternative explanation for our findings—independent of the theory of longitudinal dissociation of the His bundle [23, 24, 25]—suggests that the lesions induced by TAVR occur at the level of the branching portion of the His bundle. Given the anatomical position of this region and its close proximity to the aortic valve, it is particularly susceptible to mechanical injury, which may account for the development of escape rhythms. Damage to this branching area, including the origin of the RBB, may also explain the observed patterns of escape rhythms.

Lastly, it is noteworthy that in all 8 patients who exhibited both AV junctional and ventricular escape rhythms, the initial rhythm documented was consistently the AV junctional rhythm. This temporal pattern suggests that the emergence of a ventricular escape rhythm may reflect instability or failure of the preceding AV junctional escape rhythm.

### 4.3 Correlation Between CAVB, the Type of Valve, and Patient Sex and Age

Most (63.8%) of our study patients received an SEV, while the remaining 323 (36.7%) received a BEV. Although the total number of SEVs and BEVs implanted in the whole TAVR population is unknown, these results are consistent with the higher likelihood of developing conduction disturbances with SEV as opposed to BEV [26]. In contrast, patient age and sex were not found to have a significant influence on the site of AVB or the type of presumed escape rhythm during CAVB.

### 4.4 Study Limitations

First, this was a retrospective study, and post-TAVR ECGs were not recorded systematically. Continuous 12-lead ECG monitoring may have revealed additional or sequential escape rhythms, potentially clarifying whether ventricular rhythms were preceded by AV junctional rhythms—thus supporting His bundle involvement.

Second, ECG 2 was unavailable in approximately 20% of patients, limiting accurate classification of escape rhythm origin. In these cases, comparisons between ECG 1 and ECG 3 were used as a surrogate.

Third, EPS were not performed, preventing definitive localization of the conduction block. As such, the true prevalence of AV junctional escape rhythms may be underestimated. Additionally, our classification system may have misclassified some junctional rhythms (e.g., RBBB morphology) as ventricular.

Fourth, only patients with stable escape rhythms permitting 12-lead ECG documentation were included. Therefore, escape rhythms occurring during the TAVR procedure itself—particularly unstable or transient ones—may differ in mechanism. Finally, ECG findings were not correlated with cardiac CT imaging, which might have provided further anatomical insight into the site of conduction system injury [27].

### 4.5 Clinical Implications

Characterizing the escape rhythm during CAVB after TAVR is not merely descriptive but can help guide the choice of permanent pacing modality. The majority of patients in our analysis either had a ventricular escape rhythm (48%), and even in presumed cases of junctional origin, almost 90% displayed either LBBB or RBBB escape morphologies, with the remaining also showing conduction system abnormalities. This near-universal presence of intraventricular conduction disturbances indicates that the mechanical injury from TAVR predominantly involves the distal His bundle and bundle branches, areas that may not be effectively captured by His bundle pacing alone. His bundle pacing is limited by lower success rates in infra-nodal block (76% vs. 93% for nodal block), higher and less stable thresholds, as well as technical challenges in lead implantation and fixation. Left bundle branch area pacing (LBBAP) may offer a higher likelihood of restoring near-normal conduction and avoiding chronic right-ventricular dys-synchrony, with procedural success rates approaching 93%, and favorable sensing and pacing parameters across the spectrum of AV conduction disease, including LBBB and infra-Hisian block.

The temporal evolution of escape rhythms provides additional support for LBBAP. Ten percent of our cohort demonstrated progression from junctional to ventricular escape rhythms, indicating advancing conduction system damage from proximal to distal sites. Given that delayed progression to advanced AV block (>48 hours post-TAVR) is well-documented, reliance on proximal pacing sites may prove inadequate as conduction disease advances. Thus, LBBAP offers greater reliability. Importantly, even in cases presumed to have a ventricular escape rhythm, the site of block may still lie near the His bundle, and such patients could also benefit from conduction system pacing [11, 12, 28].

## 5. Conclusions

Analysis of ECG characteristics during CAVB provided insights into the likely site of conduction block following TAVR. Consistent with anatomical regions commonly affected by the prosthetic valve, at least 40% of patients exhibited escape rhythms suggestive of an AV junctional origin. These findings represent a working hypothesis derived from retrospective data and warrant confirmation through prospective studies incorporating electrogram recordings from the proximal conduction system, including the His bundle and its branches.

## Availability of Data and Materials

The datasets used and analyzed during the current study are available from the corresponding author on reasonable request.
